# KE and EE Genotypes of ICAM-1 Gene K469E Polymorphism Is Associated with Severe Preeclampsia

**DOI:** 10.1155/2014/124941

**Published:** 2014-01-30

**Authors:** Ehsan Tabatabai, Saeedeh Salimi, Milad Mohammadoo-khorasani, Minoo Yaghmaei, Mojgan Mokhtari, Farzaneh Farajian Mashhadi, Anoosh Naghavi

**Affiliations:** ^1^Cellular and Molecular Research Center, Zahedan University of Medical Sciences, Zahedan, Iran; ^2^Department of Clinical Biochemistry, School of Medicine, Zahedan University of Medical Sciences, Zahedan, Iran; ^3^Department of Obstetrics and Gynecology, School of Medicine, Zahedan University of Medical Sciences, Zahedan, Iran; ^4^Department of Pharmacology, School of Medicine, Zahedan University of Medical Sciences, Zahedan, Iran; ^5^Department of Medical Genetics, Faculty of Medical Sciences, Tarbiat Modares University, Tehran, Iran

## Abstract

*Background.* Preeclampsia (PE) is one of the most important complications of pregnancy that is associated with significant mortality and morbidity in mother and fetus. Since the etiologic factors in its development are still unclear, we aimed to examine the intercellular adhesion molecule-1 (ICAM-1) gene K469E polymorphism in preeclamptic and control healthy women. *Materials and Methods.* Genetic polymorphism was analyzed in 192 PE and 186 healthy control women. PCR-RFLP method was used to identify K469E polymorphism. *Results.* The frequency of KK, KE, and EE genotypes of ICAM-1 gene was not different between PE patients and healthy pregnant women. Whereas, the frequency of KE and EE genotypes was significantly higher in severe PE than mild PE women and control group, and the risk of severe PE was 2.4-fold higher in subjects with KE genotype (OR, 2.4 [95% CI, 1 to 5.9]; *P* = 0.03) and 3.3-fold higher in subjects with EE genotype (OR, 3.3 [95% CI, 1.2 to 9]; *P* = 0.015) compared to individuals with KK genotype. *Conclusion.* We concluded that KE and EE genotypes of K469E polymorphism could increase risk of severe PE.

## 1. Introduction

Preeclampsia (PE) is a pregnancy syndrome that can affect virtually every organ system with a prevalence that varies from 5% to 10% [1]. It is clinically characterized by new onset of proteinuria and hypertension after 20 weeks of gestation and associated with significant mortality and morbidity in mothers and fetuses [2]. The pathophysiological mechanisms proposed for it are complex and include placental ischemia, inflammatory pathway, oxidative stress, the renin-angiotensin system, activation of thrombosis, and genetic factors [3]. A common clinical sign of PE is endothelial dysfunction; however, the specific factors initiating endothelial dysfunction in PE are not clear. Although the etiology of PE has not been elucidated, family studies have suggested that genetic factors play an important role in its etiology [4].

Intercellular adhesion molecule-1 (ICAM-1) is involved in the pathogenetic mechanisms responsible for immune-mediated diseases including disorders of female reproductive system such as endometriosis, ovarian stimulation syndrome, and preeclampsia [5–7]. ICAM-1 is a transmembrane glycoprotein and expressed in endothelial cells and leukocytes in the immune system [8, 9]. It plays an important role in cell-to-cell interactions and participates in inflammatory processes by facilitating leukocyte endothelial transmigration at site of inflammation [10]. The ICAM-1 gene is located in 19p13.2 and its polymorphisms have been suggested to have functional activity [11, 12]. The ICAM-1 gene has two single-base polymorphisms which are common genetic variations associated with diseases: Glycine or Arginine at codon 241 of exon 4 (G241R) and Lysine or Glutamine at codon 469 of exon 6 (K469E) [13].

Association of K469E polymorphism (rs5498: A > G) with several inflammatory disorders has been studied [14, 15]. The K469E single nucleotide polymorphism located three bases upstream of the splicing site which affects the ICAM-1 mRNA splicing pattern [15–17]. The studies which detected the effect of ICAM-1 polymorphism on the risk of PE are yet outspread. However, previous studies have shown that ICAM-1 gene K469E polymorphism did not correlate with PE [18].

In the present study, we aimed to examine the association between K469E polymorphism at exon 6 of the ICAM-1 gene and preeclampsia in south east of Iran.

## 2. Subjects and Methods

### 2.1. Subjects

Subjects were recruited from the Department of Obstetrics and Gynecology of Ali-ebn-Abitaleb Hospital (Zahedan, Iran) from 2012 to 2013. Ethical committee approval was received, and informed consent was obtained from patients and control women. The study included 192 women with preeclampsia (aged 27.5 ± 7 years) and 186 unrelated healthy controls (aged 26.8 ± 6.4 years). Demographic characteristics of these subjects are presented in Table 1. PE was defined according to clinical findings including increased blood pressure (≥140 mmHg systolic or ≥90 mmHg diastolic on 2 or more measurements at least 6 h apart) and proteinuria ≥0.3 g/24 h or ≥+1 on a urine dipstick after 20 weeks of gestation [19]. Exclusion criteria included twin or multiple pregnancies or any evidence of previous medical disease. Women who are affected by systemic, infectious, cardiac, and renal diseases and systemic lupus erythematosus were excluded. The healthy state of control group was determined by medical history. None of PE patients and healthy controls had any prior history of hypertension.

Early onset PE is usually defined as preeclampsia that develops before 34 weeks of gestation, whereas late onset PE develops at or after 34 weeks of gestation. Severe PE was defined as either severe hypertension (SBP > 160 mmHg or DBP > 110 mmHg) or severe proteinuria (2 g protein in a 24 h urine collection).

### 2.2. Genotype Analysis

The DNA analysis was performed in the Cellular and Molecular Research Center (Zahedan, Iran). Genomic DNA was extracted from peripheral blood leukocytes of women with PE and controls using salt phenol chloroform method and stored at −20°C until analysis. The total volume of the polymerase chain reaction (PCR) mixture was 25 *μ*L and contained 200 ng genomic DNA, 25 pM of each primer, 0.1 mM dNTP, 1.5 mM MgCl_2_, 2.5 *μ*L PCR buffer 10x, and 1U of Taq polymerase. The forward primer was 5′-GGA-ACC-CAT-TGC-CCG-AGC-3′ and the reverse primer was 5′-GGT-GAG-GAT-TGC-ATT-AGG-TC-3′ [20]. Amplification was carried out in a Bio-Rad thermal cycler using a thermal profile of initial denaturation at 96°C for 6 min, followed by 30 cycles at 96°C for 30 s, annealing at 61°C for 30 s and primer extension at 72°C for 60 s, and a final extension step at 72°C for 6 min. The 223 bp PCR product was digested by BstU1 (Bsh1236I) restriction enzyme and was incubated at 37°C overnight. After digestion, PCR products were identified by electrophoresis on 2.5% agarose gel. The E allele has one BstU1 cleavage site and digested to 136 and 87 bp fragments, whereas the K allele has no cleavage site and produces 223 bp fragment only.

### 2.3. Statistical Analysis

Statistical analyses were performed by using SPSS18 software. Frequency of the genotypes and alleles between cases and controls were compared using the chi-square or Fisher exact tests. Quantitative variables was compared using student's *t*-test. *P* value of <0.05 was considered statistically significant. Odds ratios (OR) and 95% confidence intervals (CI) were accounted to significant allelic and genotyping associations.

## 3. Results

Demographic data of the patients and controls are presented in Table 1. No difference in the maternal age and birth weight was observed between two groups. PE women had significantly higher systolic and diastolic blood pressures than controls. Also, Gestation age and primiparity were significantly different between cases and controls (*P* < 0.05). The frequencies of three ethnic groups (Baloch, Fars, and Afghan) were significantly different between case and control groups (*P* = 0.003), and the risk of PE was 1.7-fold greater in Afghan women in comparison with Baloch and Fars women. (OR, 1.7 [95% CI, 1 to 2.8]; *P* = 0.003).

The distribution of K469E polymorphism genotypes and alleles between two groups was summarized in Table 2. The distribution of K469E polymorphism genotypes was in Hardy-Weinberg equilibrium for cases and controls. There was a similar genotypes distribution between PE and control women. Also as shown in Table 3, there was no difference in genotypes distribution between early onset and late onset preeclampsia. Nevertheless, the frequency of KE and EE genotypes was significantly higher in severe than mild PE and controls. As shown in Table 4, the risk of severe PE was 2.4-fold higher in subjects with KE genotype than subjects with KK genotype compared to controls (OR, 2.4 [95% CI, 1 to 5.9]; *P* = 0.03). Moreover, the risk of severe PE was 3.3-fold higher in subjects with EE genotype than subjects with KK genotype compared to controls (OR, 3.3 [95% CI, 1.2 to 9]; *P* = 0.015).

Furthermore, there were no minor allele frequency differences between the three ethnic groups (*P* = 0.3).

## 4. Discussion

Preeclampsia is an idiopathic multisystem disorder that is characterized by hypertension, proteinuria, and systemic inflammatory response. The origin of PE may depend on maternal constitutional factors such as obesity, genetic, dysfunctional maternal clearance, or inflammatory system; however, the exact pathogenesis of PE is still unknown [21].

ICAM-1 is an essential mediator of inflammation pathway and plays an important role in movement of leukocytes during inflammatory responses. In normal pregnancies, activated blood leukocytes initiate later inflammatory responses that are grater in PE women [22]. ICAM-1 K469E polymorphism could contribute to the functional modification of the gene product and may influence its function. Many studies illustrated associations between ICAM-1 K469E polymorphism and peripheral occlusive arterial disease [23], inflammatory bowel disease [14], coronary artery disease [24], diabetes mellitus [25, 26], and endometriosis [27]. Abnormality in the ICAM-1 protein and/or soluble ICAM-1 (sICAM-1) levels may interfere with normal immune function and increase the risk of immune-related diseases [14]. There are several studies that observed higher sICAM-1 levels in PE women compared to normotensive pregnant women [28, 29]; however, this elevation has not been documented by others [30, 31]. Indeed, Kim et al. reported elevated sICAM-1 levels only in severe preeclampsia [32].

Although inflammation-mediated changes in ICAM-1 expression and function have been seen in some PE studies [32, 33], current study did not confirm any association between genotypes or alleles of K469E polymorphism and risk of PE. Similar results were found by Lim et al., Freemen et al., and Kwon et al. [18, 34, 35]. The first study performed in United Kingdom did not show any significant association between the K469E polymorphism and PE [34]. In another study, no association was observed between the K469 allele of ICAM1 gene and PE in a Korean population [18]. A recent report from Korea included 42 PE patients and 138 controls showed that the frequency of K allele and KK genotype was higher in PE patients than controls, but there were no significant differences. They detected similar trends between the severe PE patients and controls [35]. In our PE population, the frequencies of the KE and EE genotypes were significantly higher in severe than mild PE (*P* < 0.05, Table 4). Therefore, it seems that the E allele was significantly associated with increased risk of severe PE. Moreover, there was no significant difference in K469E polymorphism genotypes and alleles in early onset and late onset preeclampsia in present study. Since determination of the plasma ICAM-1 concentration was not carried out, we could not estimate the correlation between genotypes and plasma ICAM-1 levels.

To the best of our knowledge, this was the first study about the association between ICAM-1 K469E polymorphisms and PE susceptibility in Iran.

There are some limitations in the present study for example, low sample size especially in severe PE and early onset PE groups, environmental conditions, and different ethnic groups existing in south east of Iran. Therefore due to the relatively small number of PE patients and the racial differences, further investigations using a larger sample size and different ethnic groups to confirm the present findings are necessary.

In conclusion, the results indicate that ICAM-1 gene K469E polymorphism is not a risk factor in PE pathogenesis in Iranian population. However KE and EE genotypes could play a main role in PE susceptibility.

## Figures and Tables

**Table 1:** Demographic characteristics of PE patients and controls.

Variable	Controls *n* = 186	Cases *n* = 192	*P* value	OR (95% CI)
Maternal age (years)	26.7 ± 6.4	27.5 ± 7	NS	
Gestation age (days)	269.4 ± 18	260 ± 24.5	0.001	
Birth weight (g)	2932 ± 486	2801 ± 23	NS	
SBP	114 ± 9	143.7 ± 22	0.0001	
DBP	71.3 ± 11.5	90.8 ± 13.9	0.0001	
Primiparity, *n* (%)	57 (30)	84 (44)	0.003	1.9 (1.2–2.8)
Family history of PE, *n* (%)	61 (33)	77 (40)	NS	
Race, *n* (%)				
Baloch	81 (43.5)	81 (42)	Ref = 1	
Fars	70 (37.6)	52 (27)	0.13	0.7 (0.5–1.1)
Afghan	35 (18.8)	59 (31)	0.003	1.7 (1–2.8)

NS: not significant.

**Table 2:** Genotype and allele frequencies of the K469E polymorphism in PE patients and controls.

	Control *n* = 186	Case *n* = 192	*P* value	OR (95% CI)
Genotype, *n* (%)				
KK	56 (30)	50 (26)		Ref = 1
KE	96 (52)	105 (55)	0.2	1.3 (0.8–2)
EE	34 (18)	37 (19)	0.3	1.3 (0.7–2.2)
Allele *n* (%)				
K	208 (56)	205 (53)		Ref = 1
E	164 (44)	179 (47)	0.2	1.1 (0.9–1.5)

**Table 3:** Comparison of genotypes frequency of the K469E polymorphism in women with early and late onset preeclampsia.

Genotype *n* (%)	Early PE *n* = 44	Late PE *n* = 148	*P* value	OR (95% CI)
KK	12 (27.3)	38 (25.7)		Ref = 1
KE	24 (54.5)	81 (54.7)	0.5	0.9 (0.4–2.1)
EE	8 (18.2)	29 (19.6)	0.5	0.9 (0.3–2.4)

**Table 4:** Comparison of genotypes frequency of the K469E polymorphism in women with mild and severe PE.

Genotype *n* (%)	Severe PE *n* = 50	Mild PE *n* = 142	Control *n* = 186	Severe and mild PE		Severe PE and control	
				*P* value	OR (95% CI)	*P* value	OR (95% CI)
KK	7 (14)	43 (30.3)	56 (30)		Ref = 1		Ref = 1
KE	29 (58)	76 (53.5)	96 (52)	0.04	2.3 (0.9–5.8)	0.03	2.4 (1–5.9)
EE	14 (28)	23 (16.2)	34 (18)	0.01	3.8 (1.3–10.5)	0.015	3.3 (1.2–9)
